# Synthesis and Characterization of Novel Fe-Mn-Ce Ternary Oxide–Biochar Composites as Highly Efficient Adsorbents for As(III) Removal from Aqueous Solutions

**DOI:** 10.3390/ma11122445

**Published:** 2018-12-03

**Authors:** Xuewei Liu, Guogang Zhang, Lina Lin, Zulqarnain Haider Khan, Weiwen Qiu, Zhengguo Song

**Affiliations:** 1 College of Land and Environmental, Shenyang Agricultural University, Shenyang 110866, China; liuxuewei0471@163.com; 2 Agro-Environmental Protection Institute, Ministry of Agriculture of China, Tianjin 300191, China; linlina91@163.com (L.L.); zulqarnainhaiderkhan@gmail.com (Z.H.K.); 3 College of Life Sciences, Tianjin Normal University, Tianjin 300387, China; zangguogang@163.com; 4 The New Zealand Institute for Plant and Food Research Limited, Private Bag 4704, Christchurch 8140, New Zealand; weiwen.qiu@plantandfood.co.nz

**Keywords:** Fe-Mn-Ce ternary oxide–biochar composite, characterization, arsenic, adsorption

## Abstract

The widespread pollution of water bodies with arsenic (As) necessitates the development of efficient decontamination techniques. To address this issue, we herein prepare Fe-Mn-Ce ternary oxide-biochar composites (FMCBCs) using impregnation/sintering methods and examined their physicochemical properties, morphologies, and As(III) removal performances. The specific surface area of FMCBCs increased with increasing Ce content and enhanced the quantity of surface functional groups (–OH, –COOH). The adsorption of As(III) on FMCBCs was well represented by pseudo-second-order kinetics, and the As(III) adsorption capacity of the best-performing FMCBCs (8.47 mg g^−1^ for FMCBC_3_) exceeded that of BC by a factor of 2.9. At pH = 3, the amount of adsorption of As(III) by FMCBCs reached a maximum, and the increased ionic strength could enhance adsorption capacity of FMCBCs. Moreover, an As(III) removal efficiency of ~99% was observed for FMCBC_3_ at a dosage of 8 g L^−1^, which highlighted its great potential as an absorbent for As(III) removal from contaminated water.

## 1. Introduction

The rapid progress of industrialization in the past decades has resulted in severe pollution of the environment by heavy metals [1,2]. For example, industrial activities such as metal plant operation, textile production, and mining can result in the release of heavy metal-contaminated wastewater into water bodies [3,4]. In particular, mining activities are often accompanied by the discharge of acid mine drainage that often contains toxic metals (e.g., As or Fe) and has a low pH, thereby adversely affecting the environment and human health [1,5,6,7,8,9]. Arsenic is considered to be one of the most toxic pollutants in aqueous solutions due to toxicity, bioaccumulation trends, and threats to human life and environment [10]. Among the large number of known As removal methods such as coagulation, adsorption, and ion exchange, adsorption has been recognized as the most promising one in view of its simplicity, low operation cost, and technical flexibility [11]. Moreover, adsorption is the main process influencing the migration, residual concentration, and bioavailability of As compounds in water [12,13], which highlights the need to develop novel high-performance adsorbents (e.g., those based on biochar (BC)) for As(III) removal.

BC is commonly produced by pyrolysis of organic waste and features the advantages of high porosity, large specific surface area, and unique structure that allow it to be employed as an effective adsorbent for environmental remediation [14], as exemplified by the large number of recent studies on the adsorption of heavy metals by BC. Nevertheless, the efficiency of BC-promoted As removal from aqueous solutions still needs to be improved, e.g., by hybridization of BC with Fe-Mn oxides [15,16], which feature the advantages of large surface area, high surface charge, strong adsorption capacity, and the ability to oxidize the difficult-to-remove As(III) to As(V) [17]. However, the practical applicability of Fe-Mn oxide particles is limited by their tendency to aggregate, poor pore structure, and insufficient mechanical strength. To solve these problems and increase the As(III) removal efficiency, the above mixed oxide can be combined with other materials to afford composites.

China possesses large resources of rare earth metals such as Ce [18]. Ce hydroxide is resistant to heat, acids, and alkali; and is chemically stable and has a high and selective adsorption capacity for As(III) and As(V). Zheng et al. [19] demonstrated that Ce compounds such as CeO_2,_ and Ce-Fe oxides are capable of highly selective As(III) and As(V) adsorption at a wide range of pH values. Therefore, this study was built on the results of our previous research and focused on evaluating the efficiency of As removal by Fe-Mn-Ce ternary oxide–BC composites (FMCBCs), aiming to (i) prepare a range of FMCBCs and evaluate their physicochemical properties, (ii) investigate the As(III) adsorption performance of FMCBCs in aqueous solutions, and (iii) elucidate the corresponding mechanism.

## 2. Materials and Methods 

### 2.1. Chemicals

Cerium carbonate hydrate (Ce_2_(CO_3_)_3_) and KMnO_4_ were purchased from Shanghai Yuanye Bio-Technology Co., Shanghai, China. Fe(NO_3_)_3_·9H_2_O was sourced from Shanghai Macklin Biochemical Co., Shanghai, China. Standard solutions of As(III) (1000 mg L^−1^) were obtained from Sigma-Aldrich (St. Louis, MI, USA). Ultrapure water was further purified to a resistance of 18 MΩ cm using a Millipore-Q water purification system (Burlington, MA, USA). 

### 2.2. Preparation of FMCBCs

BC was prepared by anaerobic pyrolysis of air-dried and ground corn stalk leaves in a corundum crucible under a flow of nitrogen (600 cm^3^ min^–1^) in a muffle furnace (SX-10-13, Wei Ye Experimental Instrument Co., Ltd., Tianjin, China) at 600 °C for 2 h. The obtained solid was allowed to cool to 25 °C, washed with deionized water to neutrality by the filter bottle, oven-dried, ground, and sieved through a 100-mesh nylon screen.

To prepare FMCBCs, BC (5.0 g) was weighed in the corundum crucible and treated with 0.06 M Fe(NO_3_)_3_ (40 mL), 0.24 M KMnO_4_ (40 mL), and Ce_2_(CO_3_)_3_ (1–5 g). After mixing, the slurry was ultrasonicated (Shanghai Hao Electronic Technology Co., Ltd., Shanghai, China) for 2 h, stirred, and evaporated to dryness in a constant-temperature (99 °C) water bath. The resulting residue was placed into a muffle furnace and subjected to 0.5 h anaerobic pyrolysis in a flow of nitrogen (600 cm^3^ min^−1^) at 300 °C. Ce oxide–modified BC (CBC), Fe-Ce oxide–modified BC (FCBC), Mn-Ce oxide–modified BC (MCBC), and Fe-Mn oxide-modified BC (FMBC) composites were prepared using the same method.

The prepared FMCBCs featured theoretical BC:Fe:Mn:Ce weight ratios of 24:2:3:4 (FMCBC_1_), 24:2:3:8 (FMCBC_2_), and 24:2:3:10 (FMCBC_3_).

### 2.3. Characterization of FMCBCs and BC

The Brunauer–Emmett–Teller (BET) specific surface areas and Barrett–Joyner–Halenda pore size distributions were determined from N_2_ adsorption-desorption isotherms using an automated gas sorption instrument (Nova 2000e; Quantachrome Instruments, Boynton Beach, FL, USA), and crystalline phases were identified by X-ray diffraction (XRD; Philips Electronic Instruments, Amsterdam, Netherlands). Surface morphologies were observed by scanning electron microscopy (SEM; Merlin, Zeiss, Oberkochen, Germany). The surface functional groups were characterized by Fourier-transform infrared spectroscopy (FTIR, Agilent Technology Co., Ltd., Santa Clara, CA, USA). X-ray photoelectron spectroscopy (XPS) measurements were performed on a Thermo Scientific ESCALAB 250 spectrometer (Manchester, UK) using Al Kα radiation (1486.8 eV), and data processing and peak fitting were performed using the XPSPEAK 4.1 software package (Hong Kong, China). Ash content was determined from the weight of the residue remaining after 6-h combustion of BC at 800 °C [20]. Typically, 1-g FMCBC samples were heated in a muffle furnace at 800 °C until the termination of smoke evolution and cooled to 25 °C.

### 2.4. Adsorption Kinetics

All adsorption experiments were performed at 25 ± 0.5 °C. Typically, 2.5 g samples were placed into a 500 mL beaker containing 0.01 M NaNO_3_ and 500 µL of an As(III) solution with a concentration of 100 mg L^−1^. The resulting suspensions were magnetically stirred, and samples withdrawn every 1 min for 12 h and analyzed by atomic fluorescence spectrometry (AFS-9760, Beijing Haiguang Instrument Co., Ltd., Beijing, China) after dilution according to the adsorption capacity of FMCBCs.

BC, FMCBC_1_, FMCBC_2_, and FMCBC3 samples (0.04, 0.08, 0.12, 0.16, and 0.2 g) were placed into a 50 mL brown bottle and treated with a solution of As(III) (20 mL, 20 mg L^–1^) in 0.01 M NaNO_3_. The effect of pH on adsorption was investigated by adjusting the pH in the range 3–7 using 0.10 M NaOH and HNO_3_ solutions. The effect of solution concentration on adsorption was investigated by using 0.001, 0.01, and 0.1 M NaNO_3_. The obtained suspensions were shaken for 6 h and filtered through Whatman No. 42 filter paper, and the concentrations of As(III) in the resulting filtrates were determined by atomic fluorescence spectrometry. All experiments were performed in triplicate.

### 2.5. Effect of pH and Ionic Strength

All adsorption experiments were carried out in 50 mL brown bottles, and 0.1 g dose of FMCBC_3_ were put into each bottle. The effect of pH and ionic strength on As(III) adsorption was evaluated in taking from solutions with 10, 20, 40, 60, and 80 mg L^−1^ initial As(III) concentrations in the pH range of 3–7 and different ionic strength (0.001, 0.01 and 0.1 M L^−1^ of NaNO_3_). All experiments were performed in triplicate.

### 2.6. Reusability

After FMCBC_3_ adsorbed As(III), which were washed with 0.1 M mL^–1^ NaOH as the eluent by the filter bottle [21], freeze-dried (Shanghai Dongfulong Technology Co., Ltd., Shanghai, China), re-preparing FMCBC_3_ for adsorption As(III). The experiment was repeated as it was, and it took 4 cycles (the 0.1 g dose of FMCBC_3_ were added to brown bottles containing 20 mg L^–1^ As(III)). The concentrations of As(III) in the resulting filtrates were determined by atomic fluorescence spectrometry. 

### 2.7. Data Analysis

Statistical analyses were conducted using SPSS 21.0 software (SPSS Inc., Chicago, IL, USA). An ANOVA test was used to determine significant differences between treatments, with P-values of less than 0.05 indicating statistical significance.

## 3. Results and Discussion

### 3.1. Physicochemical Properties of FMCBCs

In view of the fact that specific surface areas are generally positively correlated with adsorption performance, we initially focused on evaluating the specific surface areas of FMCBCs (Table 1). BC particle size is commonly negatively correlated with the number of surface micropores, and pore size is also negatively correlated with specific surface area.

The surface properties of particles commonly investigated using the Brunauer-Emmett-Teller (BET) method, which allows one to determine parameters such as specific surface area, porosity, and pore volume. The specific surface area of FMCBC_3_ was shown to exceed those of other FMCBCs but was still smaller than that of BC (Table 1), in agreement with the results of Lin et al. [14]. This finding was rationalized by the fact that the rough and uneven surface of FMCBCs featured BC pores which were filled with metal oxide particles, resulting in a decrease of a specific surface area. Although specific surface area is not the only factor determining the ability of FMCBCs to adsorb As, large specific surface areas are believed to enhance the above ability. Generally, the larger the specific surface area of the material, the more the surface adsorption sites, the stronger the adsorption of heavy metals, which is consistent with the results of Lin et al. [14]. The ash content of FMCBCs exceeded that of BC by ~20%, as expected (Table 1). The highest ash content was observed for FMCBC_3_, which also exhibited the highest As(III) adsorption capacity, in agreement with previous reports [22]. 

**Figure 1:**
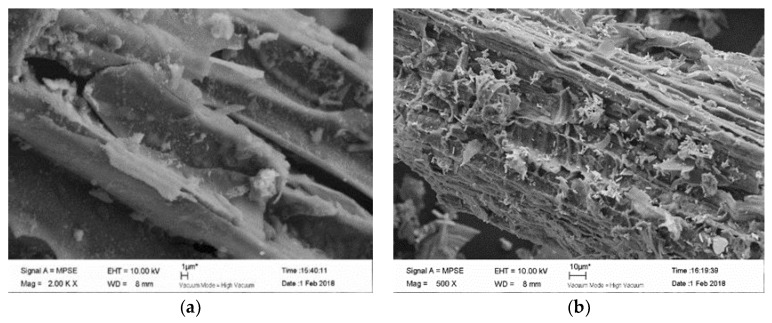

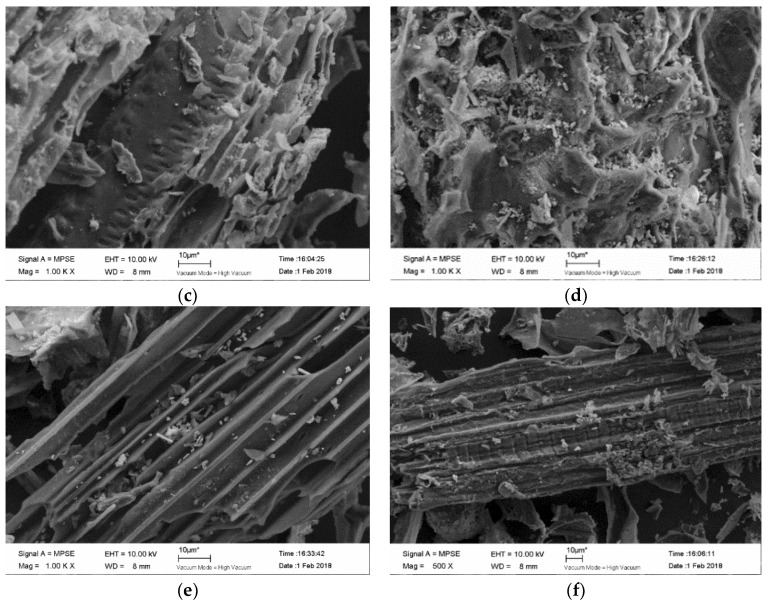
SEM analysis of the absorbents: (**a**) FMCBC_1_; (**b**) FMCBC_1_-As(III); (**c**) FMCBC_2_; (**d**) FMCBC_2_-As(III); (**e**) FMCBC_3_; (**f**) FMCBC_3_-As(III).

Figure 1 illustrates the As(III)-loaded-FMCBC_1_, As(III)-loaded-FMCBC_2_, and As(III)-loaded-FMCBC_3_ and reveals that (i) BC and FMCBCs had markedly different surface textures and morphologies, and (ii) As adsorption decreased the dimensions of surface pores. This result was explained by the formation of porous structures in FMCBCs and the concomitant increase of specific surface area and adsorption capacity upon wastewater treatment [23]. The hollow porous structure of FMCBCs provided internal sorption sites in addition to external ones, which resulted in an enhanced As(III) adsorption capacity compared to that of BC.

Figure 1a shows that while FMCBCs retained the carbon skeleton structure of their plant precursor, and their channels featured a regular block-structured surface covered by debris. This finding indicated that Fe-Mn-Ce oxide was successfully loaded on the surface of BC and confirmed that (i) the utilization of the dipping method avoided reunion effects, and (ii) the number of As adsorption sites on FMCBCs increased concomitantly with the As(III) adsorption capacity of these composites. Additionally, the presence of a large number of attachments on the surface of FMCBC_1_ demonstrated that this composite was able to adsorb As(III) from aqueous solutions (Figure 1b). Furthermore, Figure 1 reveales that other composites also retained the carbon skeleton structure of their plant precursor, and their surfaces were shown to contain traces of chemical reagents used for BC modification (Figure 1c) and featured numerous particle agglomerates. SEM imaging revealed that FMCBC_3_ exhibited a more regular structure than other composites and featured a greater number of surface-deposited attachments and particle agglomerates, which was reflected in increased specific surface area and a structure best suited for As(III) adsorption. Notably, a significant gap reduction was observed for As(III)-loaded-FMCBCs, as these gaps were filled by the adsorbed As species. Table 1 listed the elemental compositions of the utilized adsorbents, revealing the presence of carbon-attached Ce, Mn, and Fe.

**Figure 2:**
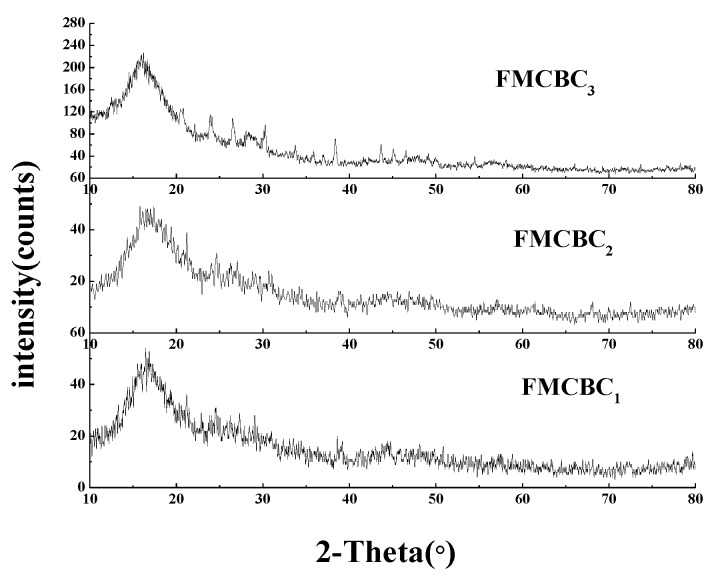
The X-ray diffraction pattern of FMCBC_s._

The XRD patterns of FMCBCs (Figure 2) featured typical diffraction peaks of crystalline Na_2_Mn_3_O_7_ and Fe_3_Al_2_(SiO_4_)_3_ at 15.7° and 17.2°, respectively, and the highest degree of crystallinity was observed for FMCBC_3_. When raw BC was combined with Fe-Mn-Ce oxide, the characteristic peak intensities of FMCBC_1_ and FMCBC_2_ were significantly reduced, which was ascribed to the disordered loading of oxides on BC in these composites. Zhang et al. [24] showed that iron, manganese and lanthanum did not have obvious crystallization peaks in the diffraction pattern, indicating that the oxides prepared in the test were all present in an amorphous form. Notably, no diffraction peaks of Ce^3+^ compounds were observed in any case, which indicates that all Ce^3+^ ions were incorporated into other crystalline phases. In addition, the intensity of the above XRD peaks increased with increasing Ce dosage, illustrating that this parameter influenced the crystallinity of Na_2_Mn_3_O_7_ and Fe_3_Al_2_(SiO_4_)_3._ At the same time, the incorporation of Ce during FMCBCs preparation could break the regular crystal structure of the iron-based material into micro-crystals, which increased the activity of FMCBCs and reduced the extent of metal leaching compared to the case of amorphous structure. 

Table 2 lists the surface atomic compositions of FMCBCs, revealing that the carbon content of these composites was lower than that of BC. However, compared to BC, FMCBCs featured an increased oxygen content, which implied that the adsorption of As(III) on these composites could potentially be accompanied by oxidation reactions. The largest carbon content reduction (from 75 to 38.82%) and the largest oxygen content increase (from 15.3 to 48.94%) were observed for FMCBC_3_. After the adsorption of As(III), the relative content of Fe decreased and that of Ce increased, i.e., arsenate was mainly adsorbed at Fe atoms, which greatly reduced the number of available Fe atoms on the adsorbent surface. In contrast to FMCBCs, no Fe, Mn, or Ce could be detected in BC, which confirmed that these elements were successfully loaded onto the surface of the above composites, with the highest loading observed for FMCBC_3_.

**Figure 3:**
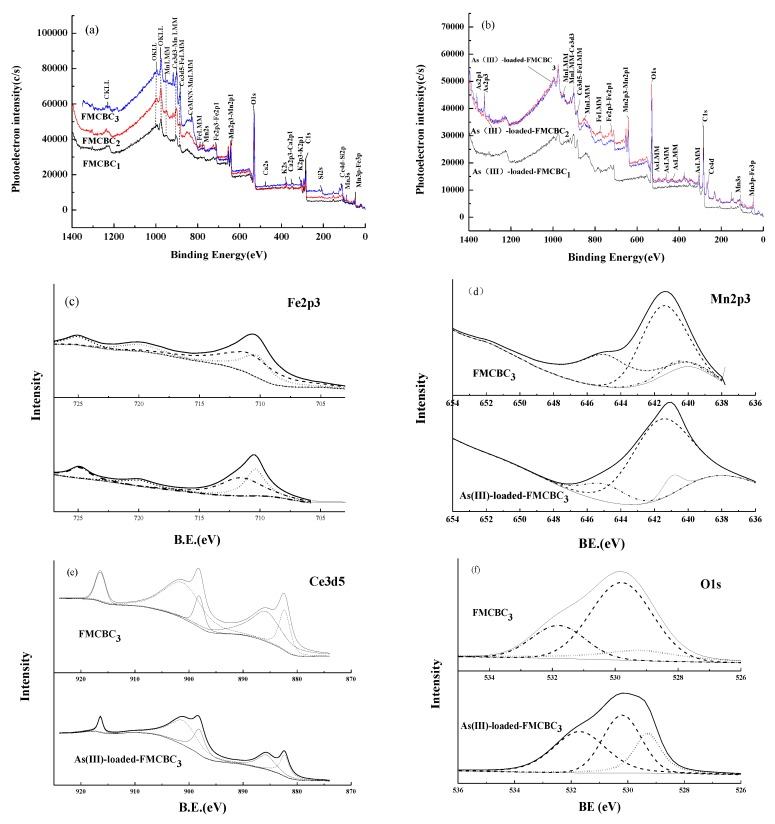
XPS analysis of FMBC with pre- and post As(III) adsorption The X-ray ((**a**) the XPS spectra of FMCBCs; (**b**) the XPS spectra of As(III)-loaded-FMCBCs; (**c**) Fe2p; (**d**) Mn2p3/2; (**e**) Ce3d5; (**f**) O1s the spectra of FMCBC_3_).

To explore the structural characteristics of FMCBCs, these composites were characterized by XPS before and after As adsorption (Figure 3). As a result, Fe, Mn, and Ce peaks were observed in all cases (Figure 3a,b), i.e., the corresponding oxides were successfully embedded into BC, changing its physicochemical properties and increasing its As(III) adsorption ability. The XPS peaks at 710.0 and 712.5 eV observed in the Fe spectrum were ascribed to Fe(II) and Fe(III), and those at 710.4 and 725.0 eV were assigned to the main and satellite peaks of Fe_2_O_3_ (Figure 3c), which confirmed that FMCBCs efficiently removed As(III) from water, which is consistent with the findings of Li et al. [25]. XPS analysis also confirmed that the Fe atoms played a key role in As adsorption while the main role for Ce atoms was to form an amorphous structure [26]. The characteristic peaks at 640.9, 641.6, and 645.0 eV were ascribed to MnO, Mn_2_O_3_, and MnO_2_, respectively (Figure 3d), which revealed the presence of Mn(II), Mn(III), and Mn(IV) on the surface of FMCBCs [27]. According to the research of Cui et al. [28], Mn(IV) can be incorporated into Fe, which is beneficial to improve the efficiency of removing As(III). The presence of strongly oxidizing MnO_2_ was thought to facilitate the adsorption of As(III) by promoting its oxidation to more easily adsorbed As(V). In aqueous solutions, the small coordination number to Mn in MnO_2_ allows it to easily form hydroxylated surfaces that bind As(III) and subsequently oxidize it to As(V) [29]. Thus, when the concentration of As(V) in the solution increased, it was desorbed into the solution and then adsorbed by carbon-based materials. Peaks at 882.3 and 915.0 eV corresponded to Ce(IV), and those at 884.0, 885.6, and 902.1 eV indicated the presence of Ce(III). Based on the intensities of these peaks, the Ce(III) content of FMCBCs exceeded that of Ce(IV). Finally, the peak at 529.2 eV was ascribed to CeO_2_, which was believed to exhibit catalytic activity by providing reactive surface lattice oxygen atoms that were consumed to form oxygen vacancies. Figure 2c demonstrates that after the adsorption of As(III), the content of Ce(IV) in FMCBC_3_ was significantly reduced, which indicates that Ce(IV) was consumed during the oxidation of As(III) to As(V), it is because tetravalent cerium ions can be heated in aqueous solution to obtain cerium oxide, which is a strong oxidizing agent and can oxidize As(III) into As(V). The released oxygen atoms could potentially oxidize activated BC, change its pore structure, and widen pores, thus increasing the specific surface area of FMCBCs and enhancing their adsorption performance.

**Figure 4:**
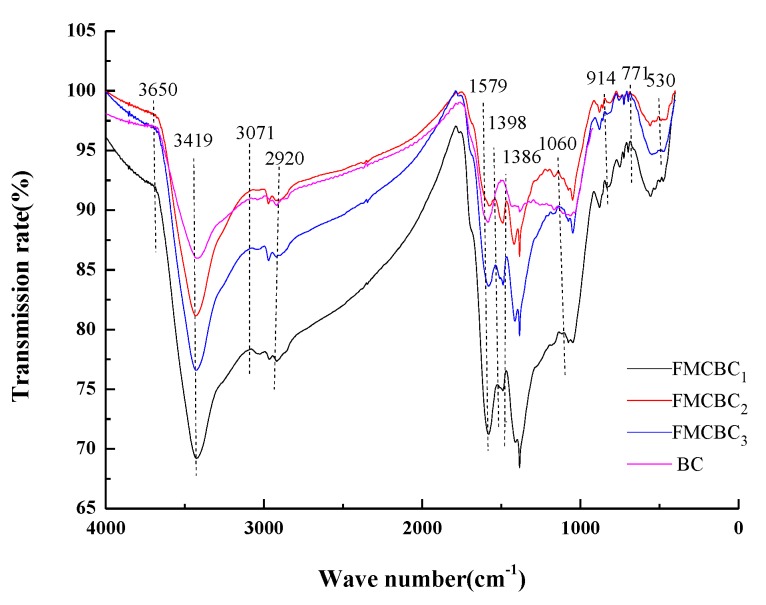
The FTIR spectrogram of FMCBCs.

The surface functional groups of FMCBCs were probed by FTIR spectroscopy. The spectra of FMCBCs and BC are fairly similar (Figure 4), and the spectra of all FMCBCs featured strong absorption peaks at identical wavenumbers. However, the intensity and width of these peaks were subject to variations caused by differences in the number of functional groups. Broad absorption bands at 3000–3500 cm^–1^ were observed, and peaks at 3650 and 3419 cm^–1^ were assigned to –OH stretching vibrations of water molecules, which the result was similar to previous research [30]. The peak at 3071 cm^−1^ was ascribed to –C=C–H vibrations, and the stretching vibration of –CH_2_– units was observed at 2920 cm^−1^. Peaks at 1800–1300 cm^−1^ were ascribed to stretching vibrations of C=O groups and aromatic ring [31]. In particular, the peak at 1579 cm^−1^ observed for FMCBCs and BC could be assigned to C=O groups of aldehydes, lactones, ketones (asymmetric stretch), or/and carboxyl groups [32]. Bands at 2929.4 and 914 cm^−1^ represented the presence of aliphatic C–H groups [33]. In general, the surface functional groups (C–H, –OH, –COOH) of FMCBCs have a strong affinity for As(III) and can therefore capture it via surface complexation and group exchange [34]. The peak at 1492 cm^−1^ was attributed to the bending vibration of Ce–OH units, and peaks at 771, 556, 530, and 419 cm^–1^ were attributed to Si–O, Fe–O, Mn–O, and Ce–O moieties, respectively, which showed that Fe-Mn-Ce oxide was successfully loaded on the surface of FMCBCs. Thus, compared to BC, FMCBCs featured Fe-, Mn-, and Ce-containing surface functional groups that could potentially form complexes with As(III), thereby enhancing its adsorption [15]. At constant pH, FMCBC_3_ was predicted to exhibit the greatest capacity for heavy metal adsorption among the prepared composites, since its surface exhibited the highest density of Fe–O, Mn–O, and Ce–O moieties [35].

### 3.2. As(III) Removal Efficiencies of FMCBCs

**Figure 5:**
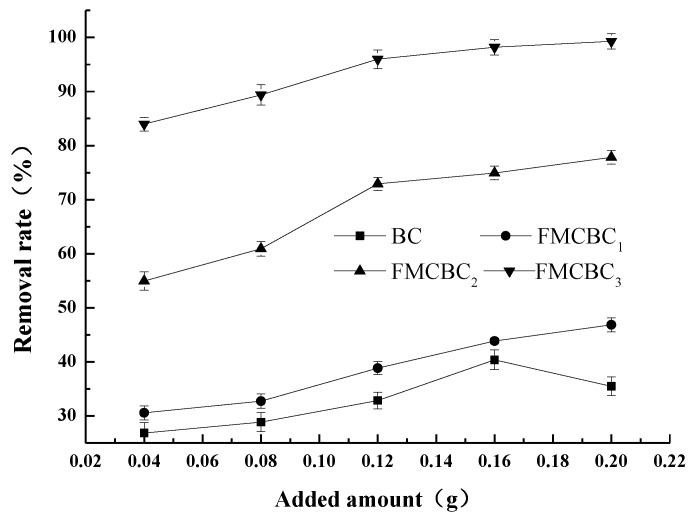
Removal rate of FMCBC_1_, FMCBC_2_ and FMCBC_3._

Figure 5 compares the As(III) removal efficiencies achieved for three different loadings of BC and FMCBCs, demonstrating that the above efficiency increased with increasing adsorbent dosage. At constant dosage, the As(III) removal efficiency depended on the type of adsorbent and was highest for FMCBC_3_. Since high adsorbent dosages inevitably increase the cost of water treatment, a balance needs to be found between the removal efficiency of As(III) and the amount of utilized adsorbent. The As(III) removal efficiency observed for an FMCBC_3_ dosage of 6 g L^−1^ exceeded 90% but did not substantially increase when the dosage was increased to 10 g L^−1^. Therefore, 6 g L^−1^ was determined as the optimal dosage. 

As mentioned above, the prepared FMCBCs contained MnO_2_ that could oxidize As(III) to As(V), and the removal of As(III) by FMCBCs was believed to involve redox reactions and not merely correspond to a simple adsorption process. Although Ce oxide is particularly well suited to remove As from water by absorption, it is much more efficient for removing As(V) than As(III), i.e., As removal efficiency can be improved by oxidizing As(III) to As(V). Compared to BC, FMCBCs released a large amount of Mn^2+^ during As adsorption, which was believed to reflect the oxidation of As(III) to As(V). Moreover, these cations were believed to adhere to the FMCBCs surface to increase its positive charge and hence facilitate the adsorption of As by electrostatic attraction and the formation of surface complexes. At the same time, the incorporation of Fe and Ce into BC significantly increased the number of active adsorption sites of the FMCBCs surface.

### 3.3. Adsorption Kinetics

The kinetics of As(III) adsorption on FMCBCs was fitted by pseudo-first- and pseudo-second-order models [36,37]. The former fitting was performed based on Equation (1):

ln(*q*_e_ − *q_t_*) = ln*q*_e_ − *k*_1_*t* (1)

where *q*_e_ and *q*_t_ (mg g^−1^) are adsorption capacities at equilibrium and time *t*, respectively, and *k*_1_ (min^−1^) is the pseudo-first-order rate constant. For the pseudo-second-order model, fitting was performed using Equation (2):

*t*/*q_t_* = 1/*k*_2_*q*_e_^2^ + *t*/*q*_e_ (2)

where *k*_2_ (g mg^−1^ min^−1^) is the pseudo-second-order rate constant.

**Figure 6:**
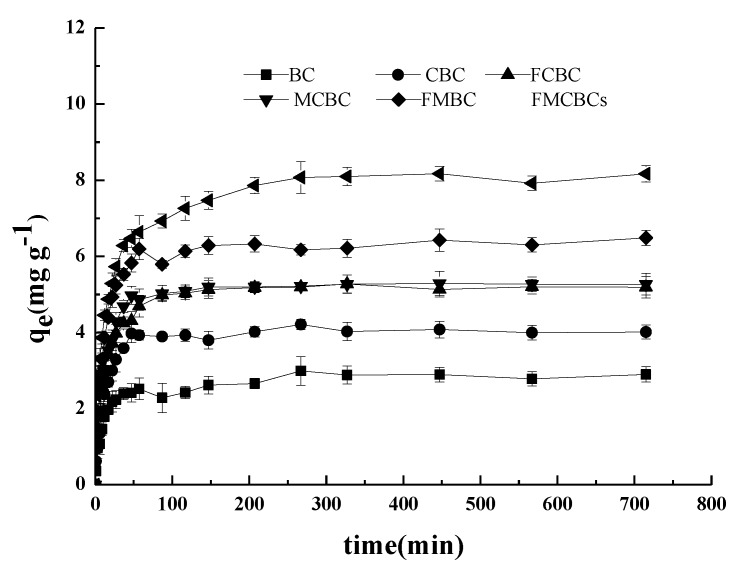
The adsorption kinetics of As(III).

Figure 6 compares the adsorption kinetics of BC and FMCBCs, demonstrating that the amount of adsorbed As(III) increased with increasing equilibrium concentration up to a certain time. This behavior was rationalized by the presence of abundant adsorption sites and the large difference between the concentrations of As in the liquid and solid phases at the initial stage of adsorption, which allowed As(III) to easily diffuse and be bound by the solid surface. Conversely, the saturation of adsorption sites resulted in a decreased adsorption rate and the establishment of an adsorption equilibrium, as reported elsewhere [38].

Figure 6 also reveals that the As(III) adsorption capacities of pristine and modified BCs dramatically increased within the first 40 min and reached maximum values (that remained stable after 200 min) after 50 min for BC, CBC, FCBC, and MCBC. In the fast adsorption phase, As(III) was believed to undergo physical adsorption by rapidly moving to nearby adsorption sites, whereas the slow adsorption phase corresponded to irreversible chemical adsorption influenced by the internal structure of modified BC [39]. Conversely, the adsorption capacities of FMCBCs and FMBC continued to increase after 50 min. The As(III) adsorption capacities of the investigated adsorbents were in the order of FMCBCs > FMBC > MCBC > FCBC > CBC > BC, with the maximum of 8.47 mg g^−1^ observed for FMCBC_3_. These results were consistent with previously reported findings [40].

Table 3 shows that the kinetics of As(III) adsorption were best fitted by the pseudo-second-order model in all cases. The fitted equilibrium adsorption amount is closer to the experimental data. This indicates that the rate-limiting step of adsorption is controlled by chemical adsorption between absorbents and As, rather than by material transfer in solution. Compared to BC, all modified materials exhibited higher As(III) adsorption capacities. 

And the sorption capacity of FMCBC exceeding those of BC, CBC, FCBC, MCBC, and FMBC by factors of 3.0, 2.14, 1.66, 1.63, and 1.34, respectively. This enhanced performance was ascribed to the change in the number and type of BC surface functional groups upon the addition of Fe-Mn-Ce oxide.

The adsorption capacity of FMCBC_3_ reached 8.47 mg g^−1^, and the sorption capacity of FMCBC_3_ far exceeded BC derived from corn straw, wheat straw, oak wood, and raw pine cone (2.84, 2.23, 1.81 and 0.0057 mg g^−1^ for As(III)) [9,41,42]. The sorption capacity of zero-valent iron-biochar complexes (4.56 mg g^−1^) for As(III)) were also lower than FMCBC_3_ [22]. The sorption capacity of FMCBC_3_ was better than FMBC that was modified biochar by potassium permanganate and ferric nitrate (8.25 mg g^−1^) [16]. 

### 3.4. Effect of pH and Ionic Strength on Adsorption

**Figure 7:**
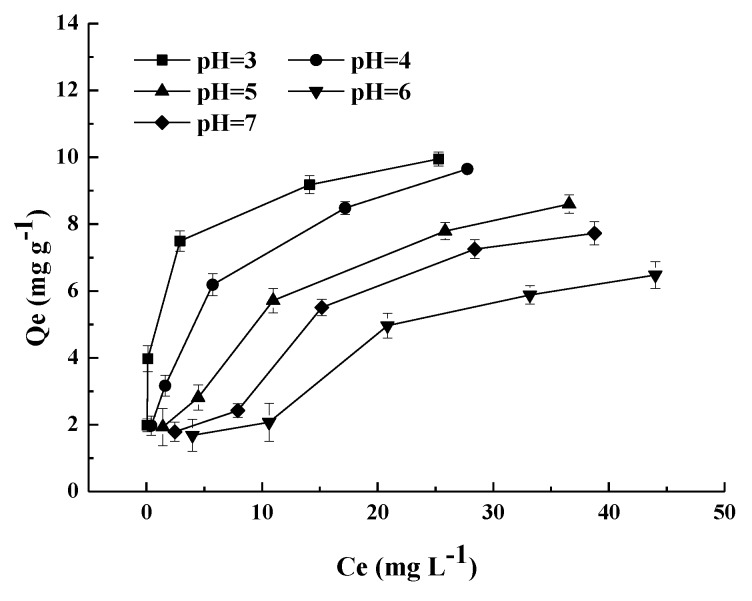
The effect of pH on adsorption by FMCBC_3_.

Figure 7 shows the influences of pH on FMCBC_3_, and the pH plays an important role in adsorbents performance on arsenic removal, since pH affects speciation of arsenic as well as characteristics of the absorbents surface such as surface charge. The effects of pH on As adsorbed by FMCBC_3_ is depicted (Figure 7), and the As removal was evidently dependent on pH with the greatest adsorption occurring at pH = 3 and then decreased with increase of solution pH till it reached pH = 7. Our results differed from those obtained in other studies [43]. Lower pH is favorable for the protonation of sorbent surface [44]. Increased protonation is thought to increase the positively charged sites, enlarge the attraction force existing between the sorbent surface and As anions, and, therefore, increase the amount of adsorption in the lower pH region.

**Figure 8:**
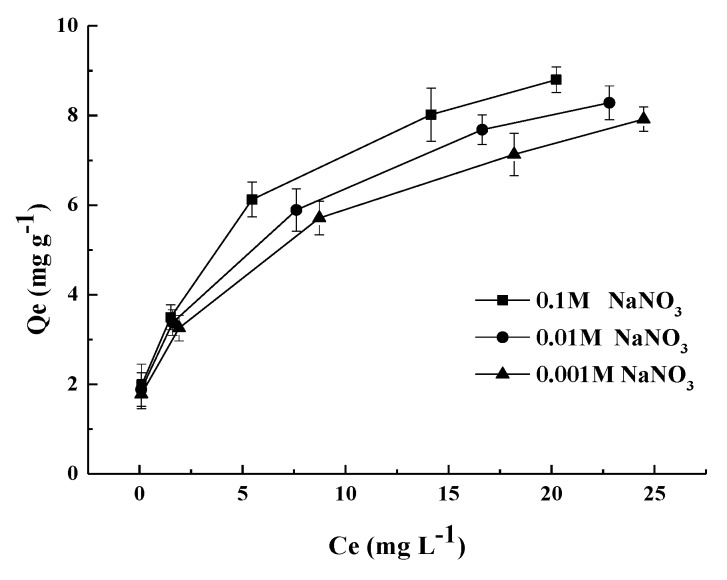
The effect of solution on adsorption by FMCBC_3_.

Figure 8 reveals that the equilibrium sorption capacities were affected by different concentration of solution NaNO_3_ (0.1 M, 0.01 M and 0.001 M), with increases of solution concentration subtly enhancing As(III) removal efficiency. The anion adsorption at the sorbent/aqueous interfaces can be classified into outer-sphere surface ion-pair complexes (weakly bonding) and inner-sphere surface coordination complexes (strongly bonding) regarding ionic strength dependence studies. It can be assumed that, FMCBC_3_ adsorbed As (III) through forming inner-sphere surface coordination complexes (strongly bonding) in which the strongly bonding anions were relatively unaffected or respond to increased concentration of background electrolyte.

### 3.5. Stability and Reusability

For efficient water treatment, the adsorbent should have good stability and regeneration characteristics. The regeneration characteristics of the adsorbent FMCBC increase the efficiency of adsorption and reduce the cost. Therefore, regeneration of FMCBC is very important for the adsorbent. Studies have shown that FMCBC has a large oxidized functional group on the surface, which has an adsorption effect on arsenic. The adsorption depends on the surface charge of FMCBC, and the surface charge is controlled by the pH of the solution. The adsorption and desorption of arsenic depends on the pH of the solution, so changes in the pH of the solution can lead to regeneration of the arsenic adsorbent [45]. We carried out a lab study to investigate As removal efficiency of regenerated FMCBC. The result was encouraging. After four cycles, the removal rate of As of FMCBC can still reach ~80% of initial concentration (Table 4).

**Figure 9:**
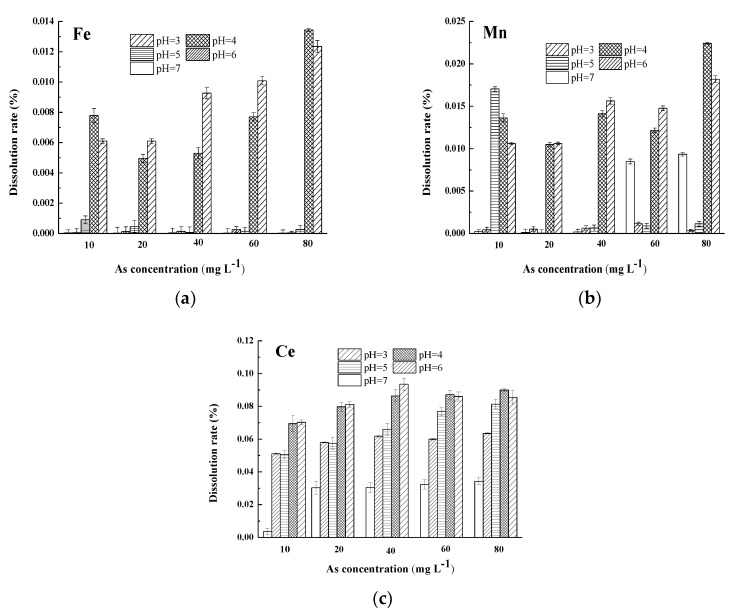
The dissolution rate of Fe (**a**), Mn (**b**), and Ce (**c**) after As(III) adsorbed by FMCBCs.

In the adsorption process, the stability of the adsorption of As by FMCBCs plays an important role in the development of adsorbents. The study showed there was a small amount of iron, manganese, and cerium ions dissolved after adsorption As(III) of FMCBCs in different As concentration. Results for pH, in Figure 9, show that the FMCBCs have excellent stability, and the arsenic in the adsorption environment has a promising potential.

## 4. Conclusions

In summary, we successfully prepared and characterized a range of FMCBCs, and then demonstrated the great arsenic removal performance on As(III) from aqueous solutions, showing that the incorporation of Fe-Mn-Ce oxide increased the number of adsorption sites on the BC surface. These modifications resulted in an increased ash content (maximum ash content and largest specific surface area among FMCBCs were observed for FMCBC_3_) and changed the internal structure of FMCBCs and the type/amount of their surface functional groups. The efficiency of As(III) removal increased with increasing FMCBCs dosage and the optimal dosage of the best-performing FMCBC_3_ adsorbent were determined as 8 g L^−1^. The adsorption of As(III) by pristine and modified BCs was well fitted by pseudo-second order kinetics, and the adsorption capacities of FMCBCs exceeded that of pristine BC. Among the three FMCBCs, FMCBC_3_ exhibited the highest As(III) adsorption capacity of 8.47 mg g^−1^ that exceeded the corresponding value of BC by a factor of 2.9. The maximum adsorption capacity of FMCBC_3_ to As(III) exceeds 10 mg g^−1^ in pH = 3, and the removal efficiency of FMCBC_3_ on As(III) was also improved with the increase of ionic strength. Therefore, FMCBCs were identified as a class of effective adsorbents suitable for As(III) removal from aqueous solutions.

## Figures and Tables

**Table 1:** Physicochemical properties of the FMCBCs.

Adsorbents	C (wt %)	N (wt %)	H (wt %)	Ash (wt %)	S_BET_ (m^2^ g^−1^)	pH
BC	85.26	0.81	5.20	10.17	60.9	8.93
FMCBC_1_	62.31	1.67	2.56	30.57	26.33	9.39
FMCBC_2_	53.34	1.71	2.47	30.66	35.74	9.61
FMCBC_3_	42.13	1.73	2.21	32.6	46.66	9.64

Note: SBET, specific surface area.

**Table 2:** The X-ray photoelectron spectroscopy of FMCBCs (%).

Adsorbents	C (wt %)	O (wt %)	Fe (wt %)	Mn (wt %)	Ce (wt %)	As (wt %)
BC	75.01	15.3	-	-	-	-
FMCBC_1_	60.04	33.7	1.2	7.44	0.64	-
FMCBC_2_	52.78	36.69	1.12	7.41	1.40	-
FMCBC_3_	38.82	48.94	1.17	8.72	1.90	-
As(III)-loaded-FMCBC_3_	37.57	44.06	1.08	5.89	2.16	1.23

Note: “-” means no detection.

**Table 3:** The parameters of dynamic fit of BC and modified materials.

Adsorbents	Pseudo First-Order			Pseudo Second-Order		
	Qe (mg g^−1^)	K_1_ (min^−1^)	R^2^	Qe (mg g^−1^)	K_2_ (mg^−1^ min^−1^)	R^2^
BC	2.83	0.0401	0.985	2.84	0.0119	0.999
CBC	4.02	0.0876	0.758	4.01	0.0113	0.999
FCBC	5.16	0.0694	0.895	5.16	0.0113	0.999
MCBC	5.26	0.0601	0.931	5.27	0.0103	0.999
FMBC	6.40	0.0435	0.854	6.41	0.0044	0.998
FMCBC	8.47	0.0464	0.632	8.45	0.0028	0.998

**Table 4:** As adsorption: cycling and regeneration of the adsorbent.

Cycle	Adsorbent (g)	As (mg/L)		Removal (wt %)
		Initial	Final	As
1	0.1	20.00	0.64	96.8
2		20.00	1.27	93.6
3		20.00	2.82	85.9
4		20.00	4.13	79.4
